# Digital Public Health – Rasanter technischer Fortschritt, aber viele offene Public-Health-Fragen

**DOI:** 10.1007/s00103-020-03092-0

**Published:** 2020-01-20

**Authors:** Hajo Zeeb, Iris Pigeot, Benjamin Schüz

**Affiliations:** 1 Leibniz WissenschaftsCampus Digital Public Health Bremen, Bremen, Deutschland; 2 Leibniz-Institut für Präventionsforschung und Epidemiologie – BIPS, Achterstr. 30, 28359 Bremen, Deutschland; 3 Fachbereich Mathematik und Informatik, Universität Bremen, Bremen, Deutschland; 4 Fachbereich Human- und Gesundheitswissenschaften, Institut für Public Health und Pflegeforschung, Universität Bremen, Grazer Straße 4, 28359 Bremen, Deutschland; 5 Fachbereich Human- und Gesundheitswissenschaften, Universität Bremen, 28359 Bremen, Deutschland

Während in den letzten Jahren in Deutschland das Thema Digitalisierung vor allem in Hinsicht auf Medizin und klinische Versorgung diskutiert wurde, nimmt die Beschäftigung mit Möglichkeiten und Limitationen digitaler Technologien im Kontext von Public Health erst jetzt deutlich Fahrt auf – eine Entwicklung, die international bereits eine wesentlich größere Dynamik sowohl in der Forschung als auch in der praktischen Umsetzung besitzt. So fand dieses Jahr schon die 9. Internationale Digital Public Health Konferenz zusammen mit der European Public Health Konferenz in Marseille statt und auch zukünftig wird Digitalisierung ein Kernthema europäischer Public-Health-Konferenzen sein.

Zurzeit kommen digitale Technologien schon in vielen Bereichen von Public Health zum Einsatz, u. a. bei der individuellen Gesundheitsförderung, der Förderung von Veränderungen im Lebensstil, z. B. durch Bewegungs-Apps, in der Gesundheitsinformation oder bei der Umweltüberwachung von Luftschadstoffen. Sie werden auch zur Verbesserung der Gesundheitsversorgung eingesetzt, u. a. in ressourcenarmen Settings. Parallel entwickelt sich eine kritische Perspektive, nach der Public Health riskiert, schlicht überschwemmt zu werden von der rasanten Entwicklung digitaler Technologien (z. B. durch die immer größer werdende Anzahl von ungeprüften und/oder schlecht evaluierten Gesundheits-Apps und Sensorgeräten).

Zu den positiven Möglichkeiten der Digitalisierung in Public Health gehört, dass viele Menschen unter geringem Kostenaufwand erreicht werden können, dass verschiedene Dimensionen effektiver Public-Health-Interventionen gleichzeitig adressierbar sind und dass große Datenmengen generiert werden können, die ein objektives Monitoring, Evaluation und Verbesserung von Programmen ermöglichen. Dem stehen mögliche Risiken gegenüber. Diese betreffen u. a. den Schutz der Privatsphäre und des geistigen Eigentums, eine unklare Wirkung auf die vorhandene sozioökonomische Ungleichheit und eine mangelnde digitale Gesundheitskompetenz. Auch eine zunehmende Abhängigkeit von Technologien und ein Verlust an persönlicher Interaktion sind kritisch zu sehen. Es fehlt zudem z. B. an Rahmenkonzepten für die Formulierung konkreter gesundheitsbezogener Bedarfe und Evidenzanforderungen an digitale Technologien. Durch die ubiquitäre Nutzung sozialer Netzwerke drohen zudem wichtige Public-Health-Themen wie Impfungen zum Spielball von Fehlinformationen und Digitaltrollen zu werden.

In der Konsequenz brauchen Public-Health-Akteure in Forschung und Praxis nicht nur klare Orientierung. Sie sind auch gefragt, ihrerseits aktiv daran mitzuarbeiten, Public Health in Zeiten der Digitalisierung sinnvoll und evidenzbasiert voranzutreiben.

In dem vorliegenden Heft vertiefen insgesamt zehn Übersichtsbeiträge die oben beschriebenen Aspekte aus verschiedenen Perspektiven. Im ersten Beitrag geben Zeeb und Co-Autoren eine grundsätzliche Einführung in das Konzept von Digital Public Health, wobei sie sowohl die Nutzung digitaler Technologien bei Public-Health-Aufgaben anhand von Beispielen als auch spezifische Herausforderungen, wie z. B. Datenschutz- und ethische Aspekte, diskutieren. In dem anschließenden Beitrag widmen sich Hochmuth et al. insbesondere den Ansätzen zur partizipativen Entwicklung und Implementierung von Interventionen mit digitalen Public-Health-Gesundheitstechnologien. Jahnel und Schüz greifen diesen Aspekt erneut auf und erläutern, wie mittels partizipativer Entwicklungsansätze unter Berücksichtigung von Bedürfnissen und Präferenzen das Spannungsfeld zwischen Evidenzbasierung im klinischen Sinne und einer praxisorientierten Evidenz aufgelöst und so Qualität und praxisrelevante Wirksamkeit verbessert werden können. Die Entwicklung und Nutzung digitaler Gesundheitskommunikation unter den Aspekten Glaubwürdigkeit und Qualitätssicherung wird im Beitrag von Salaschek und Bonfadelli diskutiert. Brockmann arbeitet den Nutzen digitaler Technologien und moderner, damit verbundener statistischer Methoden für die epidemiologische Forschung speziell im Zusammenhang mit Infektionskrankheiten heraus, wobei auch Wege aufgezeigt werden, personenbezogene Verhaltensdaten einerseits nutzbar zu machen und andererseits die Datenhoheit jeder einzelnen Person zu wahren.

Die anschließenden Beiträge setzen sich verstärkt mit den Risiken der Digitalisierung im Gesundheitsbereich auseinander. So berichten Bittlingmayer et al. zunächst den aktuellen Forschungsstand zur digitalen Gesundheitskompetenz mit Fokus auf Kinder und Jugendliche, bevor sie das Verhältnis von digitaler Gesundheitskompetenz und sozialer sowie gesundheitlicher Ungleichheit betrachten. Dass die Nutzung von digitalen Gesundheitsangeboten auch bei Erwachsenen mit soziodemografischen Faktoren assoziiert ist, arbeiten Cornejo-Müller und Co-Autoren anhand einer zusammenfassenden Bewertung einer Reihe empirischer Studien deutlich heraus. Insbesondere gibt es – bei allerdings noch limitierter Evidenzlage – zurzeit keine Anzeichen dafür, dass digitale Gesundheitsangebote zu einer Verringerung gesundheitlicher Ungleichheit führen. Im Gegenteil: Schüz und Urban zeigen in ihrer narrativen Literaturübersicht, dass sich unerwünschte Effekte digitaler Gesundheitstechnologien im Bereich Public Health auf allen Ebenen, d. h. auf individueller Ebene, auf der Ebene sozialer Beziehungen und auf Versorgungsebene, finden lassen.

Die beiden abschließenden Beiträge befassen sich insbesondere mit den ethischen und den Datenschutzaspekten von Digital Public Health. Marckmann schlägt eine ethische Kriteriologie und ein methodisches Vorgehen für die ethische Bewertung von Digital-Public-Health-Anwendungen vor. Kunz et al. weisen auf die Anforderungen des Datenschutzes hin, die von den Anbietern von Gesundheits-Apps einzuhalten sind. Bei der Umsetzung der dazu erforderlichen Maßnahmen muss das mit der Verarbeitung von Gesundheitsdaten i. d. R. einhergehende hohe Risiko für die Rechte und Freiheiten der betroffenen Personen berücksichtigt werden.

Zusammen bieten die Beiträge in diesem Heft eine public-health-bezogene Perspektive auf ein gesundheitliches und wirtschaftliches Entwicklungsfeld, das ansonsten hauptsächlich unter den Gesichtspunkten der Fortschritts- und Anwendungspotenziale für neue Technologien diskutiert wird. Dabei erweist sich Digital Public Health als ein grundlegend multidisziplinäres Forschungs- und Anwendungsfeld, in dem keine gesundheitswissenschaftliche Disziplin allein Antworten auf dringende Fragen zur Sicherung und Förderung bevölkerungsbezogener Gesundheit geben kann. Diese multidisziplinäre und gleichzeitig public-health-orientierte Perspektive erfordert natürlich auch entsprechend ausgerichtete Forschung. So konnte in Bremen im letzten Jahr mit dem „Leibniz ScienceCampus Digital Public Health“ ein Forschungsverbund realisiert werden, in dem Konzepte für Digital Public Health und nutzerorientierte Technologieentwicklung sowie Disseminations- und neuartige Evaluationskonzepte für Digital Public Health erforscht und umgesetzt werden.

Die Beiträge in diesem Heft unterstreichen die Bedeutung einer aktiven Public-Health-Forschung in diesem Bereich, um digitale Gesundheitstechnologien insbesondere in Hinblick auf konsequente Evidenzbasierung zu entwickeln und zu implementieren.

Wir wünschen Ihnen viel Freude bei der Lektüre,

Ihr Hajo Zeeb, Ihre Iris Pigeot und Ihr Benjamin Schüz

